# Outcomes After Allogeneic Hematopoietic Cell Transplantation in Adults With Myelodysplastic Syndrome With 65 Years or Older Compared to Youngers. A Retrospective Analysis of the Latin America Registry

**DOI:** 10.1111/ejh.70001

**Published:** 2025-06-26

**Authors:** Fernando Barroso Duarte, Yhasmine Delles Oliveira Garcia, Vaneuza Araújo Moreira Funke, Anderson João, Nelson Hamerschlak, Neysimélia Costa Villela, Maria Cristina Martins de Almeida Macedo, Afonso Celso Vigorito, Rodolfo Daniel de Almeida Soares, Alessandra Aparecida Paz, Mariana Stevenazzi, Lilian Diaz, Abrahão Elias Hallack Neto, Gustavo Bettarello, Breno Moreno de Gusmão, Marco Aurélio Salvino, Rodolfo Froes Calixto, Maria Cláudia Rodrigues Moreira, Gustavo Machado Teixeira, Cinthya Corrêa da Silva, Eduardo José de Alencar Paton, Vanderson Geraldo Rocha, Alicia Enrico, Carmem Maria Sales Bonfim, Ricardo Chiattone, Celso Arrais‐Rodrigues, Erika Oliveira de Miranda Coelho, Marcelo Iastrebner, Beatrice Araújo Duarte, Vergílio Antônio Rensi Colturato

**Affiliations:** ^1^ Walter Cantídio University Hospital Fortaleza Brazil; ^2^ Federal University of Ceará Fortaleza Brazil; ^3^ Federal University of Paraná Curitiba Brazil; ^4^ Amaral Carvalho Cancer Hospital São Paulo Brazil; ^5^ Albert Einstein Israelita Hospital São Paulo Brazil; ^6^ Barretos Children's Cancer Hospital São Paulo Brazil; ^7^ Brazilian Institute of Cancer Control São Paulo Brazil; ^8^ State University of Campinas UNICAMP São Paulo Brazil; ^9^ Natal Hospital Center Natal Brazil; ^10^ Clinical Hospital of Porto Alegre Porto Alegre Brazil; ^11^ Center TPH‐SMI Integral Medical Service Montevideo Uruguay; ^12^ Hospital Universitário da Universidade Federal de Juiz de Fora Juiz de Fora Brazil; ^13^ DF Star Hospital Brasília Brazil; ^14^ Beneficência Portuguesa Hospital of São Paulo São Paulo Brazil; ^15^ University Hospital Professor Edgard Santos Salvador Brazil; ^16^ Real Hospital Português Recife Brazil; ^17^ Complexo Hospitalar de Niterói Rio de Janeiro Brazil; ^18^ Hospital das Clínicas da Universidade Federal de Minas Gerais Belo Horizonte Brazil; ^19^ ONCOBIO Health Services Belo Horizonte Brazil; ^20^ Hospital das Clínicas da Universidade de São Paulo São Paulo Brazil; ^21^ Italian Hospital La Plata La Plata Argentina; ^22^ Hospital Pequeno Príncipe Paraná Brazil; ^23^ Hospital Samaritano Higienópolis‐Américas São Paulo Brazil; ^24^ Universidade Federal de São Paulo São Paulo Brazil; ^25^ Hospital Santa Joana Recife Recife Brazil; ^26^ Sanatório Sagrado Corazon Buenos Aires Argentina; ^27^ Escola Paulista de Medicina (EPM), Universidade Federal de São Paulo (UNIFESP) São Paulo Brazil

**Keywords:** allogeneic hematopoietic cell transplantation, elderly, myelodysplastic syndrome

## Abstract

This retrospective multicenter study aimed to compare outcomes and evaluate risks associated with allogeneic hematopoietic cell transplantation (allo‐HCT) in myelodysplastic syndrome patients aged ≥ 65 versus < 65 years in Latin America, across 38 transplant centers (1988–2023). Of the 441 patients, 70 (16%) were ≥ 65 years (median age 68 ± 3.7). At 5 years, overall survival (OS) was 49.3% in patients ≥ 65 vs. 56.7% in those < 65 (*p* = 0.49), and progression‐free survival (PFS) was 48.4% vs. 56.2% (*p* = 0.40). The cumulative incidence of relapse was 13.6%, and non‐relapse mortality (NRM) 33.8%. After propensity score matching, no significant differences were observed between the age groups in OS (HR = 1.16; 95% CI: 0.76–1.77; *p* = 0.488), NRM (HR = 1.03; 95% CI: 0.64–1.67; *p* = 0.90) or PFS (HR = 1.20; 95% CI: 0.779–1.83; *p* = 0.40). PFS in patients ≥ 65 years was associated with high‐risk IPSS‐R stratification (*p* = 0.0056). The age group ≥ 65 years conferred higher risk of relapse compared to patients aged < 65 years (HR = 2.77; 95% CI: 1.07–7.15; *p* = 0.035). In patients ≥ 65 years, prevalence risk was associated to male sex, reduced‐intensity conditioning, mobilized blood cells, and prior treatment, while in those < 65 years, to complications and chronic GVHD (*p* < 0.05). HCT is feasible in elderly patients. However, age may influence disease progression in very high‐risk elderly patients and risk of relapse after transplantation.

## 1 Introduction

Myelodysplastic syndromes/neoplasms (MDS) represent a heterogeneous group of clonal myeloid disorders that predominantly affect older adults, commonly defined as individuals aged 65 years or older [1]. Allogeneic hematopoietic cell transplantation (HCT) remains the only potentially curative treatment for MDS [2]. However, in patients aged 65 years and above, HCT requires a highly individualized approach due to the frequent presence of age‐related factors such as comorbidities, impaired organ function, frailty, and decreased performance status [1, 2, 3].

Chronological age alone should not serve as an exclusion criterion for HCT eligibility in elderly patients. Rather, a comprehensive evaluation, including comorbidity assessment, frailty and geriatric evaluation, disease status, and identification of transplant‐specific risks, is essential to inform treatment decisions. The development of reduced‐intensity conditioning regimens and advances in supportive care have substantially decreased transplant‐related mortality, thereby enabling a significant increase in the use of HCT among older individuals [3].

As the global population continues to age, the number of elderly patients undergoing HCT for MDS is expected to rise correspondingly [4]. Although the age at diagnosis of MDS varies geographically, Latin American countries are also experiencing demographic aging [5]. Therefore, the objective of this study was to compare transplant outcomes, including overall survival, progression‐free survival, and clinical characteristics, as well as risk factors associated with age, in patients aged ≥ 65 years versus those < 65 years who underwent allogeneic HCT for MDS in Latin America between 1988 and 2023.

## 2 Methods

### 2.1 Data Sources

All patients registered in the Latin American Registry were included in this study, comprising a total of 441 cases from 38 transplantation centers across Latin America: 34 centers in Brazil, 1 center in Uruguay, and 3 centers in Argentina. Data were collected using the electronic platform tmo.med, available at http://www.tmo.med.br, which ensures secure access through password‐protected user authentication. The data were standardized, coded, and subsequently returned to the Latin American Registry system, enabling comprehensive analysis of HCT outcomes across participating countries. All participating centers obtained approval from their respective institutional ethics committees. These private data were previously reported in the study by Duarte FB et al. [6].

### 2.2 Study Selection

This study population included all 441 consecutive patients who underwent allo‐HCT for MDS from any type of donor in Brazil (*n* = 433), Uruguay (*n* = 6), and Argentina (*n* = 2), as systematically reported to the Latin American Registry (http://www.tmo.med.br/) between January 1, 1988, and December 31, 2023. Eligibility for HCT was determined independently by each participating center, based on available diagnostic tools and local clinical decision‐making processes. For the purposes of outcome analysis, patients were stratified into two groups according to age at the time of transplantation: < 65 years (Group 1) and ≥ 65 years (Group 2). Patient confidentiality and data protection were strictly maintained throughout the study, ensuring compliance with applicable data privacy regulations.

### 2.3 Definitions

All patients with MDS were reviewed and reclassified according to the 2022 WHO classification criteria. The risk score was determined using the Revised International Prognostic Scoring System (R‐IPSS), with patients reviewed and re‐stratified accordingly. In cases where the available clinical and laboratory data were insufficient to allow re‐stratification based on the new criteria, patients were designated as ‘unclassified’. Graft sources were categorized as bone marrow (BM), mobilized blood cells or umbilical cord placental blood (UCB).

Allogeneic donors were grouped into three categories: HLA‐matched related donors, HLA‐mismatched related (including all haploidentical donors and those with a single mismatch at HLA‐A, ‐B, ‐C, or ‐DRB1), and unrelated donors (either matched HLA or mismatched).

The classification of the type of conditioning regimen was based on the agents and doses used, as follows: myeloablative conditioning (MAC) for patients receiving total body irradiation (TBI) > 500 cGy in a single exposure or > 800 cGy in fractionated exposures; busulfan > 9 mg/kg orally or ≥ 7.2 mg/kg administered intravenously (IV) or melphalan > 150 mg/m^2^ IV as a single agent or in combination with other drugs [7]. Reduced‐intensity conditioning (RIC) regimens were defined as TBI < 500 cGy in a single fraction or ≤ 800 cGy in fractionated doses, oral busulfan < 9 mg/kg or IV busulfan < 7.2 mg/kg, melphalan < 140 mg/m^2^ IV, or thiotepa < 10 mg/kg IV [8]. Regimens that did not meet criteria for MAC or RIC were classified as non‐myeloablative (NMA).

### 2.4 Statistical Analysis

Descriptive statistics were performed for variables related to patient, disease, and transplantation characteristics. In this study, we performed a survival analysis to evaluate the impact of age group on the survival of allo‐HCT patients. We used Propensity Score Matching (PSM) to control for potential selection biases, ensuring that the age groups were comparable in relation to the observed covariates. The use of PSM allows one to reduce the effect of confounding that can occur because of differences in the distribution of measured baseline characteristics between groups, control for differences in clinically relevant variables, ensuring that comparisons between age groups reflected the effect of age, rather than the influence of confounding factors [9]. After matching the propensity scores, the patients were divided into two cohorts based on age: 65 years or older and younger than 65 years at the time of allo‐HCT. The propensity score model was adjusted using relevant variables such as Sex, Race, R‐IPSS, prior Treatment, Conditioning Regimen, Molecular Testing, Donor Type, and Cell Source. We performed the matching at a 1:4 ratio, meaning that for each patient aged 65 or older, we selected four patients younger than 65 with similar propensity scores, using the “nearest” method: Matching by nearest neighbor.

After matching, the survival curve for overall survival (OS), progression‐free survival (PFS), and non‐relapse mortality (NRM) was generated using the Kaplan–Meier method and differences between patients aged < 65 years and ≥ 65 years were assessed using the log‐rank test. The impact of HCT‐CI on OS and NRM was also analyzed using the Kaplan–Meier method and log‐rank test.

Multivariable analysis was performed using Cox proportional hazards models, and competing risk regression models were developed to compare outcomes between the groups. Factors included in the analysis of risk factors for mortality and relapse risk were patient sex, age, prior treatment, graft source, type of conditioning regimen (i.e., myeloablative vs. non‐myeloablative or reduced intensity), and donor type. The cumulative incidence of relapse (CIR) and NRM were analyzed, considering them as competing risks.

To define the characteristic relapse profile of patients older than 65 years, the prevalence ratio (PR) was calculated through comparison analyses using the chi‐squared test or Fisher's exact test. Statistical analyses were performed using R Statistical Software (version 4.2.1) and SPSS v23.1, with significance set at *p* < 0.05.

## 3 Ethical Considerations

All procedures in this study followed the ethical standards of institutional review boards and national regulations, in accordance with the revised version of the Helsinki Declaration of 1975. Ethical approval for the utilization of data from the Latin American Registry of HCT for MDS for research purposes was granted by the Federal University of Ceará/Walter Cantídio University Hospital in 2025 (Conep CAAE: 57555016.5.1001.5045, Principal Investigator Dr. Fernando Barroso Duarte).

## 4 Results

Of the 441 patients included in the study, 371 (84%) were younger than 65 years, with a median age of 40 years (±17.2), while 70 patients (16%) were aged 65 years or older, with a median age of 68 years (±3.7). The baseline characteristics of all patients, stratified by age group (< 65 years and ≥ 65 years), are presented in Table 1.

**TABLE 1:** Patient and transplant characteristics (*N* = 441).

Variable	Age group	
	65 years or older (*n* = 70)	< 65 years (*n* = 371)
Patient age at transplant, median (range), year		
Patient sex		
Male	50 (71.43%)	205 (55.26%)
Female	20 (28.57%)	166 (44.74%)
*IPSS‐R*		
Low/very low risk	6 (8.57%)	35 (9.43%)
Intermediate	15 (21.43%)	85 (22.91%)
High risk	14 (20%)	73 (19.68%)
Very high risk	6 (8.57%)	22 (5.93%)
Missing data	29 (41.43%)	156 (42.05%)
HCT‐comorbidity index at transplant, *n* (%)		
0 to 1	42 (60%)	243 (65.50%)
2	5 (7,15%)	22 (5.93%)
≥ 3	11 (15.71%)	27 (7.27%)
Missing data	12 (17.14%)	79 (21.30%)
Prior treatment		
Chemotherapy	19 (27.14%)	143 (38.55%)
Hypomethylating	28 (40%)	75 (20.22%)
Chemotherapy and Hypomethylating	11 (15.71%)	22 (5.93%)
No treatment	11 (15.71%)	127 (34.23%)
Missing data	1 (1.43%)	4 (1.07%)
Conditioning regimen		
Reduced intensity	42 (60%)	72 (19.41%)
Myeloablative	19 (27.14%)	284 (76.55%)
Non‐myeloablative/	9 (12.86%)	15 (4.04%)
Donor type		
HLA‐matched related	43 (61.43%)	237 (63.88%)
HLA‐mismatched related (Haploidentical)	12 (17.14%)	43 (11.59%)
Unrelated	15 (21.43%)	91 (24.53%)
Graft source		
Umbilical cord placental blood	0 (0%)	6 (1.62%)
Bone marrow	25 (35.71%)	193 (52.02%)
Mobilized blood cells	45 (64.29%)	172 (46.36%)
Post HCT events	45 (64.29%)	299 (80.59%)
Disease progression at any time	35 (77.78%)	143 (47.82%)
CMV reactivation	16 (35.56%)	119 (39.8%)
Acute GVHD	16 (35.56%)	142 (47.49%)
Chronic GVHD	9 (20%)	110 (36.79%)
Infections*	31 (68.89%)	238 (79.6%)

*Note:* *Bacterial infections, including multidrug‐resistant bacterial infections, and fungal infections.

Abbreviations: CMV, cytomegalovirus; GVHD, graft‐versus‐host disease; HCT, Hematopoietic Cell Transplantation; HLA, Human Leucocyte Antigen; R‐IPSS, Revised International Prognostic Scoring System.

Among patients aged 65 years or older, the majority were men (71.43%). According to the IPSS‐R risk index, 15 (36.59%) were classified as intermediate risk and 14 (34.15%) as high risk. Only 31 (7%) patients underwent molecular analysis. Most patients received grafts from HLA‐matched related donors 43 (61.43%), unrelated donors 15 (21.43%), or HLA‐haploidentical donors 12 (17.14%). The Hematopoietic Cell Transplantation Comorbidity Index (HCT‐CI) was evaluated in the present study. Most patients aged 65 years or older (*n* = 42, 60%) had at transplant between 0 and 1, 11 had HCT‐CI > 3 (15.71%), 5 had HCT‐CI 2 (7.15%), and in 12 (17.14%) of the cases, data was missing (Table 1). The OS was analyzed in the entire cohort to evaluate the impact of HCT‐CI on transplant outcomes. Patients with HCT‐CI ≥ 3 showed lower OS, with a median of 3.15 years, compared to 0–1 and 2 groups, including those with missing data. However, no statistically significant difference was observed (*p* = 0.39) (Figure S1a). Additionally, NRM was analyzed according to HCT‐CI to investigate its impact on transplant‐related mortality. Patients with HCT‐CI ≥ 3 had the highest mortality incidence (35.7%), followed by those with HCT‐CI 0–1 (33.3%) and HCT‐CI = 2 (21%). Nevertheless, no statistically significant differences (*p* = 0.825) (Figure S1b).

The principal graft source was mobilized blood cells, accounting for 45 (64.29%) of cases, followed by bone marrow (BM) transplantation, which represented 25 (35.71%). Regarding prior treatment, 58 (84.06%) patients had received previous treatment, with most of them receiving hypomethylating agents 28 (40.58%) and 19 (27.54%) receiving chemotherapy. RIC was used in 42 (60%) of patients aged 65 years or older (Table 1).

A total of 45 (64.29%) patients over 65 years of age had post‐transplant complications. The most common cause of complications was disease progression 35 (77.78%), followed by infections in 31 (68.89%) (Table 1).

**FIGURE 1:**
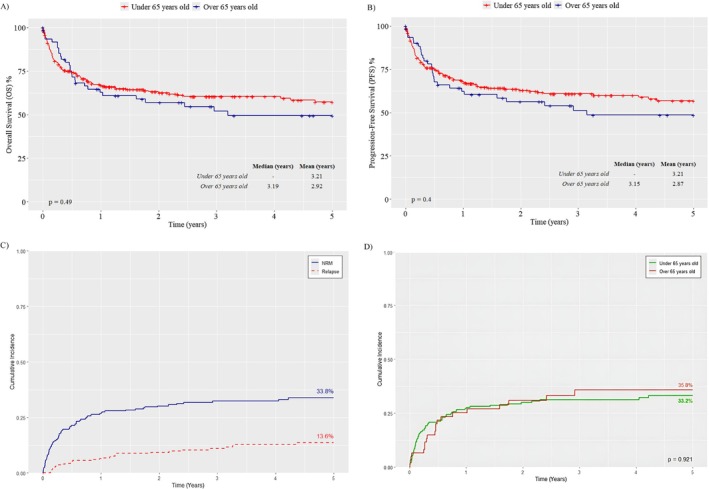
Overall survival (OS) (A), progression‐free survival (PFS) (B), cumulative incidence of relapse (CIR) and non‐relapse mortality (NRM) in patients aged 65 years or older (C) The NRM stratified by age group (panel D) (*n* = 441).

At 5 years, the OS was 49.3% for patients aged 65 years or older, with a median survival of 3.19 years, and 56.7% for patients younger than 65 years, but without statistical significance (*p* = 0.49) (Figure 1a). The estimated 5‐year PFS rate, was 48.40% for patients aged 65 years or older, and 56.20% for those younger than 65 years, with no statistical significance (*p* = 0.4) (Figure 1b). In 5‐year, the CIR was 13.6% (orange line), while NRM was 33.8% (purple line) in patients aged 65 years or older (Figure 1c). The NRM stratified by age group is demonstrated in (Figure 1d) (*p* = 0.921).

To evaluate the impact of age on the survival of allo‐HCT patients, we employed PSM to control for potential selection biases, ensuring that the age groups were comparable regarding observed covariates (Table S1). After PSM, the analysis of OS (HR: 1.16; 95% CI: 0.76–1.77; *p* = 0.49), progression‐free survival (PFS) (HR: 1.20; 95% CI: 0.779–1.83; *p* = 0.4) and Non‐Relapse Mortality (NRM) (HR = 1.03; 95% CI: 0.64–1.67; *p* = 0.921) (Figure 1d) did not show significant differences between the cohorts of patients aged over 65 years and under 65 years, respectively (Table S1).

**FIGURE 2:**
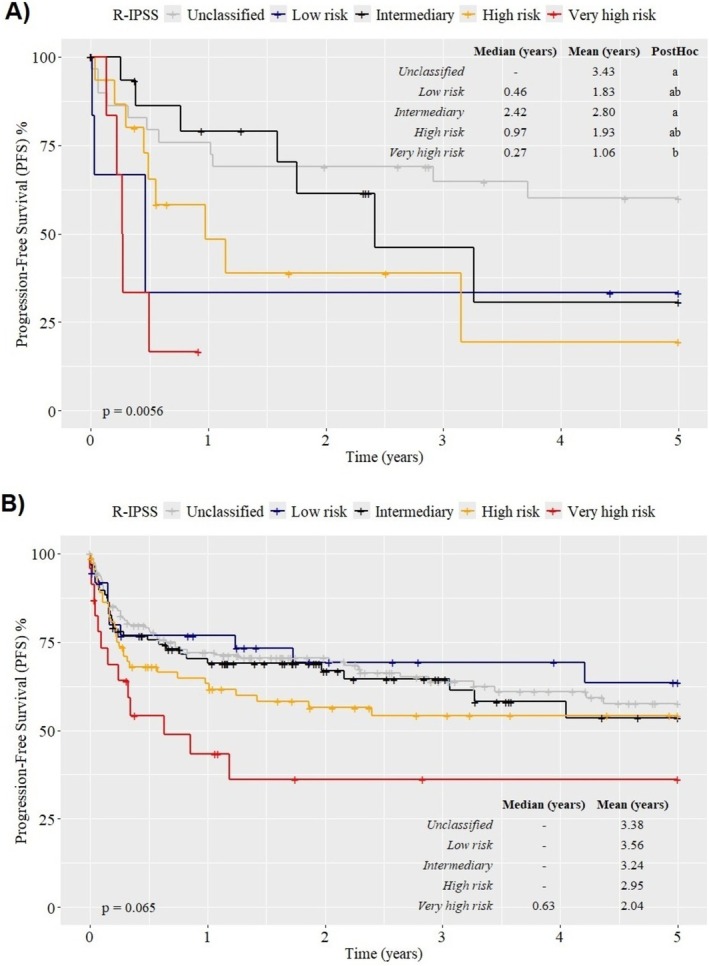
PFS after allogeneic HCT in patients aged 65 years or older (A) and younger than 65 years (B) by R‐IPSS stratification. In patients aged 65 years or older, the 5‐year PFS estimates were 33.3% for low risk (blue line), 30.7% for intermediate risk (black line), 19.4% for high risk (yellow line), 16.7% for very high risk (red line), and 59.7% for unclassified cases (gray line). The very high‐risk group showed significantly lower PFS, with a median survival of 0.27 years (*p* = 0.0056, Panel A). In contrast, no statistically significant association was observed between PFS and R‐IPSS categories in patients < 65 years (*p* = 0.065, Panel B).

**FIGURE 3:**
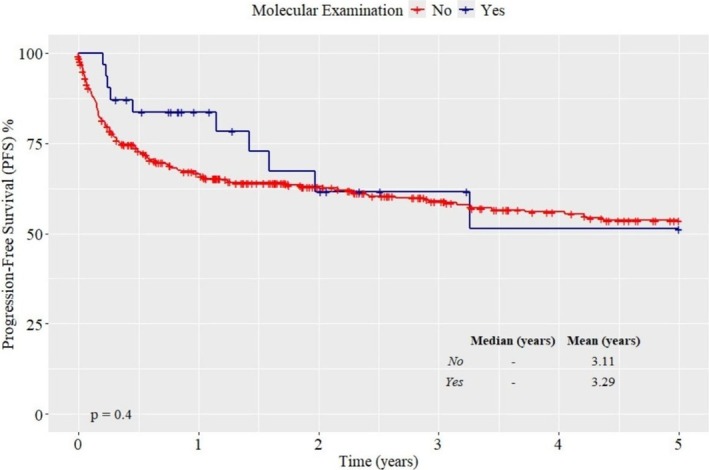
PFS according to molecular examination in patients aged 65 years or older. The 5‐year PFS estimate after HCT was 51%, with a mean survival of 3.29 years (*p* = 0.4) (*n* = 70).

As shown in Figure 3, PFS after allo‐HCT varied across the IPSS‐R risk categories at 5 years. In patients aged 65 years or older, the 5‐year PFS estimates were 33.3% for low risk, 30.7% for intermediate risk, 19.4% for high risk, 16.7% for very high risk, and 59.7% for unclassified cases. The very high‐risk group had a significantly lower PFS, with a median survival of 0.27 years (*p* = 0.0056, Figure 2A).

In patients younger than 65 years, the 5‐year PFS estimates were 63% for low risk (blue line), 52.9% for intermediate risk (black line), 54.2% for high risk (yellow line), 36.1% for very high risk (red line), and 57% for unclassified cases (gray line), but no statistically significant association between PFS and R‐IPSS categories was observed in patients < 65 years (*p* = 0.065, Figure 2B).

Molecular testing in patients aged 65 years or older, was associated with a lower PFS rate (51%), with a median survival of 3.29 years compared to patients who did not undergo testing, but without statistical significance (*p* = 0.4) (Figure 3).

The Cox regression model was used to evaluate the risk of death among patients; key variables such as gender, age group, cell source, prior treatment, conditioning regimen, and donor type were considered (Table 2). In multivariable analysis, the mobilized blood cells source was associated with a 28% lower risk of death in patients aged 65 years or older (HR, 0.72; 95% CI, 0.52–0.98; *p* = 0.040) (Table 2). Additionally, in the multivariable analysis after adjustment in the Cox model for relapse‐free survival (RFS) time, patients aged 65 years or older had 2.77‐fold higher risk of relapse compared to patients younger than 65 years (HR, 2.77; 95% CI, 1.07–7.15; *p* = 0.035) (Table 3).

**TABLE 2:** Risk factors for survival time after HCT in patients ≥ 65 years of age—Results of multivariate analysis.

Variable	HR* (95% CI)	*p*
Patient sex		
Female	1	
Male	1.21 (0.88–1.66)	0.247
Age groups, years		
Under 65 years	1	
65 years or older	1.35 (0.88–2.06)	0.172
Graft source		
Bone marrow	1	
Mobilized blood cells	0.72 (0.52–0.98)	**0.040**
Prior treatment		
No	1	
Yes	0.97 (0.69–1.36)	0.852
Conditioning regimen		
Non‐myeloablative/reduced intensity	1	
Myeloablative	1.07 (0.74–1.54)	0.721
Donor type		
Related HLA‐matched	1	
Related HLA‐haploidentical	0.92 (0.55–1.54)	0.758
Unrelated	0.88 (0.6–1.3)	0.536

*Note:* *Cox proportional hazards regression. Bold values indicate statistically significant differences (*p* < 0.05).

Abbreviations: CI, confidence interval; HLA, Human Leucocyte Antigen; HR, hazard ratio.

**TABLE 3:** Risk factors for relapse‐free survival time after HCT in patients ≥ 65 years of age—Results of multivariate analysis.

Variable	HR* (95% CI)	*p*
Patient sex		
Female	1	
Male	0.83 (0.39–1.77)	0.625
Age group, years		
Under 65 years	1	
65 years or older	2.77 (1.07–7.15)	**0.035**
Graft source		
Bone marrow	1	
Mobilized blood cells	0.5 (0.22–1.11)	0.089
Prior treatment		
No	1	
Yes	0.97 (0.42–2.24)	0.950
Conditioning regimen		
Non‐myeloablative/reduced intensity	1	
Myeloablative	0.66 (0.29–1.54)	0.341
Donor type		
Related HLA‐matched	1	
Related HLA‐haploidentical	1.14 (0.37–3.54)	0.820
Unrelated	0.36 (0.1–1.27)	0.112

*Note:* *Cox proportional hazards regression. Bold values indicate statistically significant differences (*p* < 0.05).

Abbreviations: CI, confidence interval; HLA, Human Leucocyte Antigen; HR, hazard ratio.

According on the previous results, which indicated an association between age group and relapse occurrence, a comparison of age group with other variables was performed to outline a characteristic profile of patients aged over 65. The prevalence of risk in the age group ≥ 65 years was associated with male sex (PR = 1.29; 95% CI: 1.09–1.54; [*p* = 0.012]), reduced‐intensity conditioning (PR = 3.09; 95% CI: 2.33–4.1; [*p* < 0.001]) and non‐myeloablative (PR = 3.18; 95% CI: 1.45–6.98; [*p* < 0.001]), transplantation using mobilized blood cells as the cell source (PR = 1.39; 95% CI: 1.13–1.7; [*p* = 0.021]), prior treatment (PR = 1.29; 95% CI: 1.13–1.46; [*p* = 0.002]), and type of prior treatment with hypomethylating agents (PR = 1.99; 95% CI: 1.4–2.82; [*p* < 0.001]) or chemotherapy plus hypomethylating agents (PR = 2.66; 95% CI: 1.35–5.32; [*p* < 0.001]) (Table S2). The presence of complications (PR = 0.8; 95% CI: 0.67–0.96; [*p* = 0.003]) and chronic GVHD (PR = 0.54; 95% CI: 0.3–0.99; [*p* = 0.027]) were associated with a lower prevalence of risk in patients ≥ 65 years of age compared to those younger than 65 years (Table S2).

## 5 Discussion

The results of this study showed no significant differences in OS and PFS in patients aged 65 years or older compared with younger patients. These results are consistent with a study conducted by the Center for Blood and Marrow Transplantation Research (CIBMTR), which included 688 elderly patients (> 65 years) with MDS, and found no statistically significant difference in 3‐year overall survival between elderly patients and the younger cohort (*p* = 0.06) [10].

In the present study, age 65 or older did not show a significant difference in 5‐year PFS when compared to younger patients, even after adjusting for PSM between the groups. Similarly, a study on the outcomes of allo‐HCT in adults ≥ 70 years with hematologic malignancies across the United States reported a 2‐year PFS estimate of 39% (95% CI, 35%–42%) [11].

However, patients in the very high‐risk category had the lowest 5‐year PFS estimate compared to those in lower‐risk categories. IPSS‐R classifications are used to estimate the risk of disease progression to AML, OS, and to guide therapeutic decisions in patients with MDS. Higher IPSS‐R scores at the time of transplant are associated with worse outcomes due to the severity of cytopenias, the percentage of bone marrow blasts, the presence of karyotypic abnormalities, and mutations that promote progression to AML [12, 13].

The outcome after HCT depends on cytogenetic and molecular genetic characteristics. However, no significant association was found between molecular testing and PFS according to age. Regarding the new system, the International Prognostic Scoring System for molecular (IPSS‐M), which integrates genomic profiling with hematologic and cytogenetic parameters, improves risk stratification for MDS patients, represents a valuable tool for clinical decision‐making and is more predictive than the IPSS‐R (MDS) [14]. In a predictive survival analysis comparing IPSS‐M and IPSS‐R in elderly individuals with MDS, IPSS‐M showed an advantage over IPSS‐R in older individuals, due to a higher frequency of mutations and a greater number of deleterious genes correlated with older age [15].

Although it is widely recognized that age is a risk factor for allo‐HCT outcomes, the multivariable analysis in the present study showed no significant difference in mortality between patients aged 65 years or older and those younger than 65 years. This finding is consistent with other studies in which age was not significantly associated with the risk of death in patients with myelodysplastic syndrome undergoing HCT (HR, 1.09; *p* = 0.23) [10]. Given these findings, HCT remains a viable therapeutic option for older patients undergoing allogeneic transplantation for MDS [16]. Additionally, a lower risk of mortality was observed among patients aged 65 years or older who received a mobilized blood cells graft (HR, 0.72; *p* = 0.04). In this context, some studies have also demonstrated that the use of mobilized blood cells compared to BM is associated with improved survival, especially in patients with high‐risk hematologic malignancies [17].

Relapse remains the leading cause of death in MDS patients following allo‐HCT. In the present study, we observed that age over 65 years had an impact on the risk of relapse, which is consistent with the literature, as advanced age has emerged as a strong predictor of relapse due to both an overall increased disease risk and a reduced ability to receive myeloablative conditioning because of comorbidities and functional impairment. However, conflicting results have been reported, such as in the retrospective study by Atallah et al. [10], which found no significant difference in relapse risk when comparing MDS patients aged 65 years or older with those aged 55 to 64 years (*p* = 0.07), as well as in the study by McClune et al. [18] (*p* = 0.87), where age was not significantly associated with relapse.

Given the impact of age on relapse risk, we evaluated the clinical profile of patients aged ≥ 65 years and observed that male sex, the use of a RIC or non‐myeloablative conditioning, the use of mobilized blood cells as the stem cell source, and prior treatment with hypomethylating agents were associated with a lower risk of prevalence in the ≥ 65 years age group. Regarding sex, MDS occurs more frequently in older males and in individuals with prior exposure to cytotoxic therapy [2]. A study associating sex with allo‐HCT outcomes in onco‐hematological diseases found that OS, PFS, and relapse rates were significantly worse for male recipients, regardless of the donor's sex, compared to female recipients [19]. Concerning the conditioning regimen, the development of RIC regimens has made it possible to offer HCT to elderly patients and those with comorbid conditions who are unable to tolerate traditional MAC regimens due to their considerable toxicity and high NRM. In general, studies suggest that RIC regimens are associated with an increased risk of relapse [20]. Regarding the source of stem cells, in recent years, mobilized blood cells (MBCs) have virtually replaced BM as the source of hematopoietic stem cells in allogeneic transplants. Hematopoietic recovery using this source of cells is faster after the use of MBCs compared to BM. Additionally, the minimal risk for the donor and the rapid availability are among the advantages of this source of cells [21]. However, specific patient characteristics may suggest a preference for MBCs or BM. MBCs are often preferred in individuals with a higher risk of graft failure. For instance, patients with malignant diseases who have not previously undergone cytotoxic chemotherapy present an increased risk of graft rejection and may benefit from the use of MBCs [10]. The selection of a treatment strategy before HCT for MDS is a delicate process and should focus on achieving the best survival outcomes for patients. In healthy individuals under 60 to 65 years old, with > 10% blasts in the bone marrow and no high‐risk cytogenetic abnormalities, intensive chemotherapy (ICT) should be seriously considered to reduce tumor burden before HCT. For patients up to 75 years old, therapy with hypomethylating agents should be considered before transplantation [22]. In the Latin American study on the impact of prior treatment in allo‐HCT patients with MDS, the type of prior treatment among treated patients showed a difference in OS, with treatment using hypomethylating agents, along with pre‐HCT chemotherapy, seeming to result in better survival [6].

The retrospective nature and the size of the population may introduce biases in the present study. Data, as well as information required for 2022 WHO classification and IPSS‐R stratification, were limited in the registry due to restricted access to cytogenetic and molecular testing in several participating centers.

Therefore, the decision‐making process for HCT requires a thorough evaluation and an honest discussion with patients and their caregivers to counsel them on the risks of HCT, including relapse, early mortality, complications, GVHD, and a decline in functional capacity [1]. A comprehensive geriatric assessment (CGA) could increase the likelihood of HCT success.

Future research directions should focus on identifying less aggressive yet more effective conditioning regimens for elderly MDS patients undergoing HCT. For example, the use of treosulfan‐based conditioning (FluTreo), which possesses myeloablative properties with lower toxicity and has replaced standard myeloablative regimens [1]. Although it is currently unavailable in our setting, it represents a promising option to be evaluated in this elderly population in the future [23].

We conclude that, in Latin America, allo‐HCT is feasible in elderly patients with MDS, with no significant differences in OS or mortality risk compared to younger patients. However, age may influence disease progression in very high‐risk elderly patients with MDS, as well as the risk of relapse after transplantation. We identified that male sex, the use of a reduced‐intensity conditioning regimen, and a mobilized blood cell graft are prevalent risk factors associated with elderly patients with MDS undergoing HCT.

## Author Contributions

F.B.D. contributed to the development of the concept and study design, coordinated the research, and helped write the manuscript. N.H., V.A.M.F., N.C.V., M.C.M.A.M., A.C.V., R.D.A.S., A.A.P., M.S., L.D., A.E.H.N., G.B., B.M.G., M.A.S., R.F.C., M.C.R.M., G.M.T., C.C.S., E.J.A.P., V.G.R., A.E., C..M.S.B., R.C., E.O.M.C., M.I., and V.A.R.C. diagnosed and treated the patients, provided clinical information, and contributed to patient follow‐up. A.J.S. and C.C.S. contributed to the statistical analysis. Y.D.O.G. helped write the manuscript. All authors read and approved the final manuscript. The B.A.D. contributed to the translation of this manuscript.

## Ethics Statement

Ethical approval for the utilization of data from the Latin American Registry of HCT for MDS for research purposes was granted by the Federal University of Ceará/Walter Cantídio University Hospital in 2025 (Conep CAAE: 57555016.5.1001.5045, Principal Investigator Dr. Fernando Barroso Duarte).

## Consent

All procedures in this study followed the ethical standards of institutional review boards and national regulations, in accordance with the revised version of the Helsinki Declaration of 1975.

## Conflicts of Interest

The authors declare no conflicts of interest.

## Supporting information


**Figure S1.** HCT‐CI impact on transplant outcomes (*n* = 441): (a) OS stratified by HCT‐CI at 5 years. (b) NRM stratified by HCT‐CI at 5 years. HCT‐CI, Hematopoietic Cell Transplantation–Comorbidity Index; NRM, non‐relapse mortality; OS, overall survival.

**Table S1.** Multivariate analysis survival endpoints matched cohort. HR, Hazard Ratio; CI, Confidence interval; PFS, Progression free‐survival; PSM, Propensity score matching. The PSM was performed for the variables: sex, Revised International Prognostic Scoring System (IPSS‐R), prior treatment, conditioning regimen, molecular testing, donor type, and cell source.

**Table S2.** Prevalence risk of relapse post‐transplant by age group. *p*‐value (a). Chi‐Square Test; (b). Fisher’s Exact Test. *Bacterial infections, including multidrug‐resistant bacterial infections, and fungal infections. CMV, cytomegalovirus; GVHD, graft‐versus‐host disease; HLA, Human Leucocyte Antigen; HCT, Hematopoietic Cell Transplantation; R‐IPSS, Revised International Prognostic Scoring System.

## Data Availability

Data may be available from the corresponding author upon reasonable request.
