# Supplementary figures and images for: Outcomes After Allogeneic Hematopoietic Cell Transplantation in Adults With Myelodysplastic Syndrome With 65 Years or Older Compared to Youngers. A Retrospective Analysis of the Latin America Registry
Source: Eur J Haematol. 2025 Jun 26;115(4):349–57. doi: 10.1111/ejh.70001 (PMC12402853; doi:10.1111/ejh.70001)

**Supplementary Figure**

**Figure S1:**

**a)**


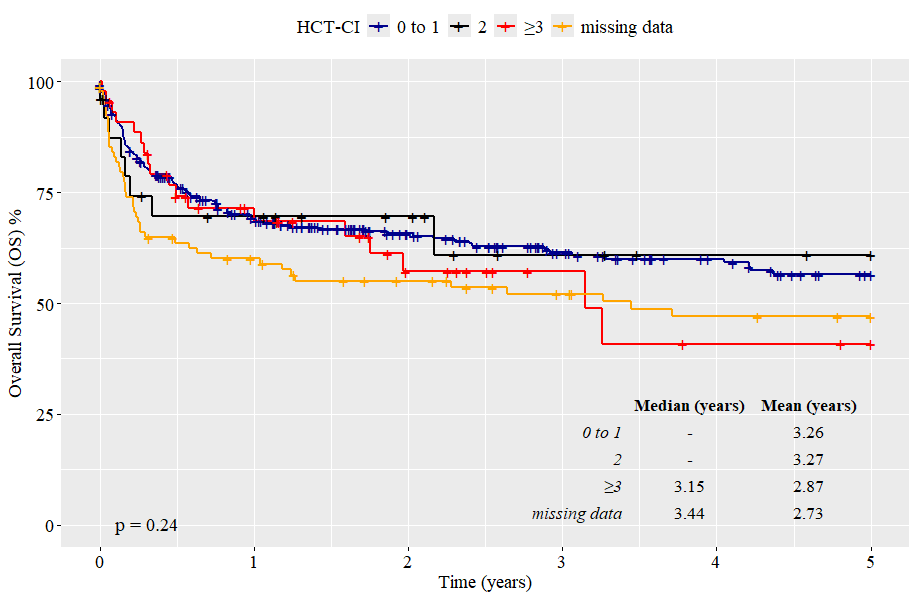


**b)**
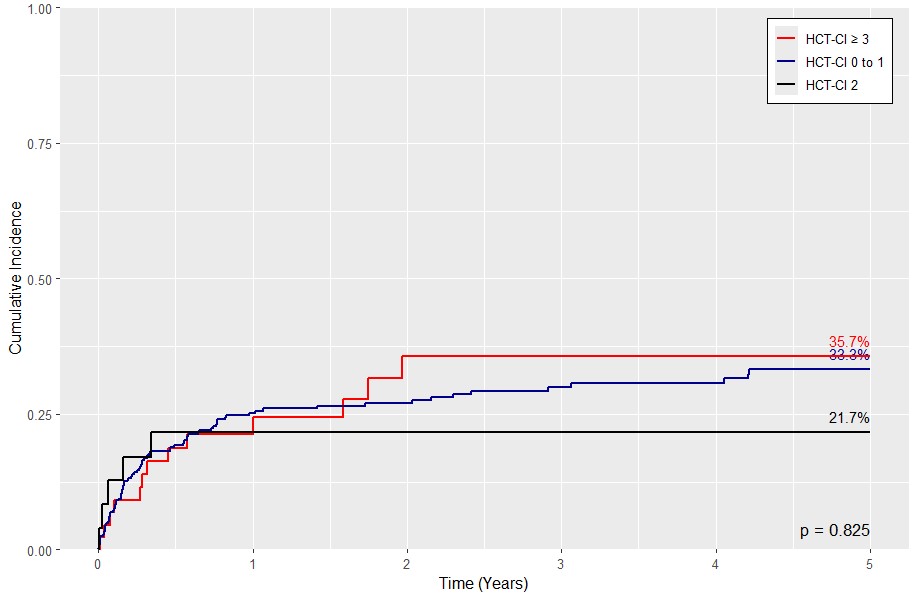

Supplement: Supplementary file 1 — Figure S1. HCT‐CI impact on transplant outcomes (n = 441): (a) OS stratified by HCT‐CI at 5 years. (b) NRM stratified by HCT‐CI at 5 years. HCT‐CI, Hematopoietic Cell Transplantation–Comorbidity Index; NRM, non‐relapse mortality; OS, overall survival. [file EJH-115-349-s002.docx]
