# Supplementary material for: Outcomes After Allogeneic Hematopoietic Cell Transplantation in Adults With Myelodysplastic Syndrome With 65 Years or Older Compared to Youngers. A Retrospective Analysis of the Latin America Registry
Source: Eur J Haematol. 2025 Jun 26;115(4):349–57. doi: 10.1111/ejh.70001 (PMC12402853; doi:10.1111/ejh.70001)
Supplement: Supplementary file 2 — Table S1. Multivariate analysis survival endpoints matched cohort. HR, Hazard Ratio; CI, Confidence interval; PFS, Progression free‐survival; PSM, Propensity score matching. The PSM was performed for the variables: sex, Revised International Prognostic Scoring System (IPSS‐R), prior treatment, conditioning regimen, molecular testing, donor type, and cell source. [file EJH-115-349-s001.docx]

**Table S1.** Multivariate analysis survival endpoints matched cohort.

| **PSM** | **OS** | | | **PFS** | | | **NRM** | | |
| --- | --- | --- | --- | --- | --- | --- | --- | --- | --- |
|  | **HR** | **95% CI** | **p-value** | **HR** | **95% CI** | **p-value** | **HR** | **95% CI** | **p-value** |
| **Analysis Group Age**  **(<65 years and ≥65 years or older)** | 1,16 | 0,76-1,77 | 0,49 | 1,2 | 0,79–1,83 | 0,4 | 1,03 | 0,64–1,67 | 0,9 |

Abbreviations: HR = Hazard Ratio, CI = Confidence interval, PFS= Progression free-survival, PSM= Propensity score matching. The PSM was performed for the variables: sex, Revised International Prognostic Scoring System (IPSS-R), prior treatment, conditioning regimen, molecular testing, donor type, and cell source.
