# Supplementary material for: Outcomes After Allogeneic Hematopoietic Cell Transplantation in Adults With Myelodysplastic Syndrome With 65 Years or Older Compared to Youngers. A Retrospective Analysis of the Latin America Registry
Source: Eur J Haematol. 2025 Jun 26;115(4):349–57. doi: 10.1111/ejh.70001 (PMC12402853; doi:10.1111/ejh.70001)
Supplement: Supplementary file 3 — Table S2. Prevalence risk of relapse post‐transplant by age group. p‐value (a). Chi‐Square Test; (b). Fisher’s Exact Test. *Bacterial infections, including multidrug‐resistant bacterial infections, and fungal infections. CMV, cytomegalovirus; GVHD, graft‐versus‐host disease; HLA, Human Leucocyte Antigen; HCT, Hematopoietic Cell Transplantation; R‐IPSS, Revised International Prognostic Scoring System. [file EJH-115-349-s003.docx]

**Table S2 –** Prevalence risk of relapse post-transplant by age group.

| **Variable** | **Age grous** | | **p value** | **PR (CI 95%)** |  |
| --- | --- | --- | --- | --- | --- |
|  | **> 65 years**  **(n =70)** | **< 65 years**  **(n = 371)** |  |  |  |
| **Patient age at transplant, median (range), year** | | |  |  |  |
| **Patient sex** | |  |  |  |  |
| Male | 50 (71,43%) | 205 (55,26%) | **0,012^a^** | **1,29 (1,09 - 1,54)** |  |
| Female | 20 (28,57%) | 166 (44,74%) |  | 1 |  |
| ***IPSS-R*** |  |  |  |  |  |
| Low/Very Low Risk | 6 (8,57%) | 35 (9,43%) | 0,846^b^ |  |  |
| Intermediate | 15 (21,43%) | 85 (22,91%) |  |  |  |
| High Risk | 14 (20%) | 73 (19,68%) |  |  |  |
| Very High Risk | 6 (8,57%) | 22 (5,93%) |  |  |  |
| Missing IPSS-R | 29 (41,43%) | 156 (42,05%) |  |  |  |
| **Prior treatment history** |  |  |  |  |  |
| Yes | 58 (82,86%) | 240 (64,7%) | **0,002**^a^ | **1,29 (1,13 - 1,46)** |  |
| No | 11 (15,71%) | 127 (34,23%) |  |  |  |
| Missing data | 1(1,43%) | 4 (1,07%) |  |  |  |
| **Prior treatment type** |  |  |  |  |  |
| Chemotherapy | 19 (27,14%) | 143 (38,55%) | **<0,001**^a^ | 0,71 (0,47 - 1,06) |  |
| Hypomethylating | 28 (40%) | 75 (20,22%) |  | **1,99 (1,4 - 2,82)** |  |
| Chemotherapy and Hypomethylating | 11 (15,71%) | 22 (5,93%) |  | **2,66 (1,35 - 5,23)** |  |
| No treatment | 11 (15,71%) | 127 (34,23%) |  | **0,46 (0,26 - 0,81)** |  |
| Missing data | 1(1,43%) | 4 (1,07%) |  |  |  |
| **Conditioning regimen** |  |  |  |  |  |
| Reduced intensity | 42 (60%) | 72 (19,41%) | **<0,001**^b^ | **3,09 (2,33 - 4,1)** |  |
| Myeloablative | 19 (27,14%) | 284 (76,55%) |  | **0,35 (0,24 - 0,52)** |  |
| Non-myeloablative/ | 9 (12,86%) | 15 (4,04%) |  | **3,18 (1,45 - 6,98)** |  |
| **Donor type** |  |  |  |  |  |
| Related HLA-matched | 43 (61,43%) | 237 (63,88%) | 0,417^a^ |  |  |
| Related HLA-haploidentical | 12 (17,14%) | 43 (11,59%) |  |  |  |
| Unrelated match | 15 (21,43%) | 91 (24,53%) |  |  |  |
| **Graft source** |  |  |  |  |  |
| Umbilical cord blood | 0 (0%) | 6 (1,62%) | **0,021**^b^ | - |  |
| Bone marrow | 25 (35,71%) | 193 (52,02%) |  | **0,69 (0,49 - 0,95)** |  |
| Mobilized blood cells | 45 (64,29%) | 172 (46,36%) |  | **1,39 (1,13 - 1,7)** |  |
| **Post HCT complications** |  |  |  |  |  |
| Yes | 45 (64,29%) | 299 (80,59%) | **0,003** ^a^ | **0,8 (0,67 - 0,96)** |  |
| No | 25 (35,71%) | 72 (19,41%) |  | 1 |  |
| **Chronic GVHD** |  |  |  |  |  |
| Yes | 9 (20%) | 110 (36,79%) | **0,027** ^a^ | **0,54 (0,3 - 0,99)** |  |
| No | 36 (80%) | 189 (63,21%) |  | 1 |  |
| Total | 45 (100%) | 299 (100%) |  |  |  |
| **Acute GVHD** |  |  |  |  |  |
| Yes | 16 (35,56%) | 142 (47,49%) | 0,134 ^a^ |  |  |
| No | 29 (64,44%) | 157 (52,51%) |  |  |  |
| Total | 45 (100%) | 299 (100%) |  |  |  |
| ***CMV* reactivation** |  |  |  |  |  |
| Yes | 16 (35,56%) | 119 (39,8%) | 0,587 ^a^ |  |  |
| No | 29 (64,44%) | 180 (60,2%) |  |  |  |
| Total | 45 (100%) | 299 (100%) |  |  |  |
| **Infections***** |  |  |  |  |  |
| Yes | 31 (68,89%) | 238 (79,6%) | 0,105 ^a^ |  |  |
| No | 14 (31,11%) | 61 (20,4%) |  |  |  |
| Total | 45 (100%) | 299 (100%) |  |  |  |
| **Disease Progression** |  |  |  |  |  |
| Yes | 35 (50%) | 143 (38,54%) | 0,073^a^ |  |  |
| No | 35 (50%) | 228 (61,46%) |  |  |  |
| Total | 70 (100%) | 371 (100%) |  |  |  |

Note: p-value a. Chi-Square Test; b. Fisher's Exact Test.*Bacterial infections, including multidrug-resistant bacterial infections, and fungal infections. Abbreviations: CMV: cytomegalovirus; GVHD: graft-versus-host disease; HLA: Human Leucocyte Antigen HCT: Hematopoietic Cell Transplantation; R-IPSS: Revised International Prognostic Scoring System.
